# Older Portuguese And Mexican Adults And Sexual Well-Being? A Cross-Cultural Qualitative Study

**DOI:** 10.1093/geroni/igab046.3275

**Published:** 2021-12-17

**Authors:** Sofia von Humboldt, Neyda Ma Mendoza-Ruvalcaba, José Alberto Ribeiro-Gonçalves, Gail Low, Isabel Leal

**Affiliations:** 1 Instituto Superior de Psicologia Aplicada (ISPA), Lisboa, Lisboa, Portugal; 2 Universidad de Guadalajara CUTONALA, Guadalajara, Jalisco, Mexico; 3 University of Alberta, Edmonton, Alberta, Canada

## Abstract

Objectives: Sexual well-being (SWB) refers to the subjective emotional and cognitive evaluation of the quality of the individual's sexuality, it plays a relevant role in quality of life and health promotion on old age and has cross-cultural implications. The aim of this study is to analyse comparatively the perspectives of older adults on their SWB in Portugal and Mexico. Methods: Data were collected from 86 Portuguese and 80 Mexican community-dwelling participants aged 65 years and older, using a semi-structured interview protocol. Older adults were inquired about their perceptions on what contributes to their sexual well-being. Socio-demographic data were also enquired. Content analysis was used to identify key themes. Results: Outcomes indicated eight themes: eroticism, supportive relationship, positive self-concept, health and self-care, romance, active life, tenderness and care, and no pain and no pregnancy restrictions, for both samples. Eroticism was the most frequent theme reported by Portuguese participants (31.4%) and health and self-care were the most frequent theme reported by Mexican participants (26.5%). Conclusions: The empirical results of this study indicated that SWB is strongly influenced by socio-cultural and psychosocial values. This cross-cultural comparison between Portugal and Mexico contributes to understand this concept in old age with different perspectives and place a scenario for future culture-adapted interventions and comprehensive policies. Keywords: Mexican, older adults; Portuguese; qualitative study; sexual well-being

